# Detection of β-amyloid aggregates/plaques in 5xFAD mice by labelled native PLGA nanoparticles: implication in the diagnosis of Alzheimer’s disease

**DOI:** 10.1186/s12951-023-01957-5

**Published:** 2023-07-10

**Authors:** Karthivashan Govindarajan, Satyabrata Kar

**Affiliations:** Departments of Medicine (Neurology), Centre for Prions and Protein Folding Diseases, Neuroscience and Mental Health Institute, University of Alberta, Edmonton, AB T6G 2M8 Canada

**Keywords:** Alzheimer’s disease, β-amyloid, PLGA nanoparticles, 5xFAD mice, Nano-theragnostic

## Abstract

Evidence suggests that increased level/aggregation of β-amyloid (Aβ) peptide, together with enhanced phosphorylation/aggregation of tau protein, play a critical role in the development of Alzheimer’s disease (AD), the leading cause of dementia in the elderly. At present, AD diagnosis is based primarily on cognitive assessment, neuroimaging, and immunological assays to detect altered levels/deposition of Aβ peptides and tau protein. While measurement of Aβ and tau in the cerebrospinal fluid/blood can indicate disease status, neuroimaging of aggregated Aβ and tau protein in the brain using positron emission tomography (PET) enable to monitor the pathological changes in AD patients. With advancements in nanomedicine, several nanoparticles, apart from drug-delivery, have been used as a diagnostic agent to identify more accurately changes in AD patients. Recently, we reported that FDA approved native PLGA nanoparticles can interact with Aβ to inhibit its aggregation/toxicity in cellular and animal models of AD. Here, we reveal that fluorescence labelled native PLGA following acute intracerebellar injection can identify majority of the immunostained Aβ as well as Congo red labelled neuritic plaques in the cortex of 5xFAD mice. Labelling of plaques by PLGA is apparent at 1 h, peak around 3 h and then start declining by 24 h after injection. No fluorescent PLGA was detected in the cerebellum of 5xFAD mice or in any brain regions of wild-type control mice following injection. These results provide the very first evidence that native PLGA nanoparticles can be used as a novel nano-theragnostic agent in the treatment as well as diagnosis of AD pathology.

**Supplementary Information:**

The online version contains supplementary material available at 10.1186/s12951-023-01957-5.

## Highlights

ICV injection of labelled native PLGA (NP) detects Aβ plaques in 5xFAD mouse brains.

NP Co-localizes well with Aβ immunostained/Congo red labelled neuritic plaques.

NP primarily stains neuritic plaque core and its peripheral regions.

NP labelled plaques peak around 3 h after injection and then declines progressively.

Labelled NP was not detected in the cerebellum of 5xFAD or in wild-type mouse brains.

**Supplementary Information:**

The online version contains supplementary material available at 10.1186/s12951-023-01957-5.

## Introduction

Alzheimer’s disease (AD), the most common type of senile dementia affecting elderly people, is characterized by the presence of tau-positive intracellular neurofibrillary tangles, extracellular β-amyloid (Aβ) containing neuritic plaques and loss of neurons in selected brain regions including basal forebrain, hippocampus, and neocortex, whereas striatum and cerebellum are relatively spared [1, 2]. Genetic, biochemical, and pathological changes associated with AD [1] indicate that an imbalance between Aβ production and removal leading to its increased levels can contribute to the loss of neurons and development of disease pathology. Historically, AD has been diagnosed based on symptomatology, with definitive diagnosis relies on post-mortem examination [3]. More recently, an update guideline from the National Institutes on Aging and the Alzheimer’s Association (NIA-AA) set out a biomarker-based framework approach towards AD diagnosis which includes Aβ deposition, tau pathology and neurodegeneration (A/T/N) [4, 5]. While the measurement of molecular biomarkers (i.e., Aβ_1−42_, total-tau and phosphorylated-tau) in body fluids may indicate disease status, neuroimaging [i.e., magnetic resonance imaging (MRI) and positron emission tomography (PET)] tests such as Aβ-PET, tau-PET, fluoro-2-deoxy-D-glucose (FDG)-PET and volumetric MRI can monitor the pathological changes in living AD patients [6, 7]. The most commonly used Aβ tracer is ^11^ C-Pittsburgh Compound-B (PiB) which exhibits high affinity, selectivity and specificity for fibrillar Aβ without specific binding to white matter. However, the cost and short half-life of ^11^ C tracers (20 min) limits their broad clinical use [8–10], whereas Fluorine-18 radiotracers with a half-life of 110 min that show analogous results to those obtained with ^11^ C-PIB, which includes ^18^ F-Florobetaben, ^18^ F-Flutemetamol, ^18^ F-Florbetapir and ^18^ F-NAV4694 that are gaining momentum for routine clinical use. The appropriate use of Aβ-PET, when combined with clinical and cognitive assessment, can significantly improve the accuracy of a clinical diagnosis of AD [10, 11]. Although the number of current molecular/imaging biomarkers has expanded recently, there is a great need for the development of novel biomarkers for a timely, precise AD diagnosis in order to provide appropriate care and treatment strategies.

Accompanying issues with early diagnosis, at present, there is no effective treatment to arrest the progression of AD. The cholinesterase inhibitors and the glutamate NMDA receptor antagonist memantine that have been approved by US Food and Drug Administration (FDA) provide symptomatic relief for only a fraction of AD patients [12]. The clinical benefits of Aβ monoclonal antibodies Aducanumab and Lecanemab remain to be determined. A major limiting factor in AD is the blood-brain barrier (BBB) preventing entry of drugs/agents into the brain [13]. Over the last decade, nanoparticles, which are engineered materials less than 100 nm in diameter with unique physicochemical properties, have been explored extensively as an area of novel therapeutic modalities to overcome the BBB. Various drugs/molecules can easily be entrapped or encapsulated into nanoparticles and then penetrate the BBB, allowing effectual drug delivery at the targeted site [14, 15]. Strategies utilizing nanoparticles that are in development for AD treatment include targeted delivery of cholinesterase inhibitors, phytochemicals, metal chelators and agents to interfere with Aβ and/or tau aggregation and toxicity [16–18].

Interestingly, acidic poly (D, L-lactide-co-glycolide) (PLGA) nanoparticles which constitute a family of FDA-approved biodegradable polymers have long been studied as delivery vehicles for drugs, proteins, and other macromolecules. These PLGA nanoparticles are synthesized from glycolic acid and lactic acid which are readily hydrolyzed in the body to produce the original monomers that are removed by the citric acid cycle as CO_2_ and H_2_O without inflicting any toxicity on cells. The degradation rate of PLGA polymer containing a 50:50 ratio of lactic and glycolic acids is much faster than those containing higher proportions of either of the two monomers [19, 20]. The excellent biocompatibility nature of PLGA have facilitated its safe use in medicinal applications since the 1970s [21]. Evidence suggests that native PLGA (without conjugation with any drug/agent), after entering cells, can traffic to lysosomes to reduce lysosomal pH, suggesting a role for native PLGA in the treatment of various diseases associated with lysosomal dysfunction including AD [22]. In fact, we recently reported that native PLGA can ameliorate not only Aβ aggregation/toxicity but also AD-related pathology in cellular and 5xFAD mouse models of AD [23, 24]. Considering the evidence that native PLGA can attenuate Aβ aggregation under in vitro conditions, it is of clinical relevance to determine if labelled PLGA can interact with Aβ aggregates of neuritic plaques in 5xFAD mouse model of AD. Using fluorescent labelled PLGA, we reveal that immunostained Aβ-/Congo red-containing neuritic plaques in 5xFAD mouse brain can be labelled with PLGA following single acute administration. In contrast, labelled PLGA did not accumulate in control wild-type mouse brains. These data suggest that native PLGA may be used not only as a potential therapeutic for the treatment of AD, but also in tracking in vivo pathological changes in living AD patients.

## Materials and methods

### Materials

Fluorescein isothiocyanate (FITC)-labelled Degradex® PLGA nanoparticles (50:50 resomer) were obtained from Phosphorex (Hopkinton, MA, USA). Alexa Fluor 594 conjugated secondary antibody and ProLong Gold anti-fade reagent were purchased from Thermo Fisher Scientific Inc. (Nepean, ON, Canada). Congo Red was procured from Sigma-Aldrich (St. Louis, MO, USA). Anti-amyloid Fibrils OC antibody and artificial cerebrospinal fluid were obtained from EMD Millipore (Burlington, MA, USA) and Harvard Apparatus (Holliston, MA, USA), respectively. All other chemicals were obtained from either Sigma-Aldrich or Fisher Scientific.

### Characterization of Fluorescence-labelled PLGA nanoparticles

The physiochemical properties of labelled PLGA such as the mean particle size, surface charge and polydispersity index (PDI) were assessed using a Malvern Zetasizer-Nano ZS (Malvern Instruments, MA, USA) equipped with a back-scattering detector (173^o^). The nanoparticle samples were prepared at 100 µg/ml in phosphate-buffered saline (0.01 M, pH 7.4) as described earlier [23, 24]. First, the samples were pre-equilibrated to room temperature and then the size and zeta potential data were determined using a 10 mm quartz cuvette filled with 150 µL sample and a disposable folding capillary cuvette filled with 1 ml sample, respectively. Three independent samples were subjected to a minimum of 10 consecutive runs of 10 s each to calculate the mean particle size, charge and polydispersity index using the manufacturer’s software (DTS v6.20).

### Animals

To determine the diagnostic potential of native PLGA targeting amyloid plaques in AD brain, we used 5xFAD mice which co-express three Amyloid precursor protein (Swedish mutation: K670N, M671L; Florida mutation: I716V; London mutation: V717I) and two Presenilin1 (M146L and L286V) Familial AD mutations and the age-matched Wild-type (WT) controls on C57BL/6J background. The phenotype and characteristic features of these 5xFAD mice have been described previously [24, 25]. These mice were purchased from The Jackson Laboratory (Bar Harbor, ME, USA) and housed on a 12 h light/dark cycle with access to food and water *ad libitum* in accordance with Canadian Council on Animal Care guidelines.

### Intracerebroventricular (icv) administration of PLGA into 5xFAD and control mice

**Fig. 1:**
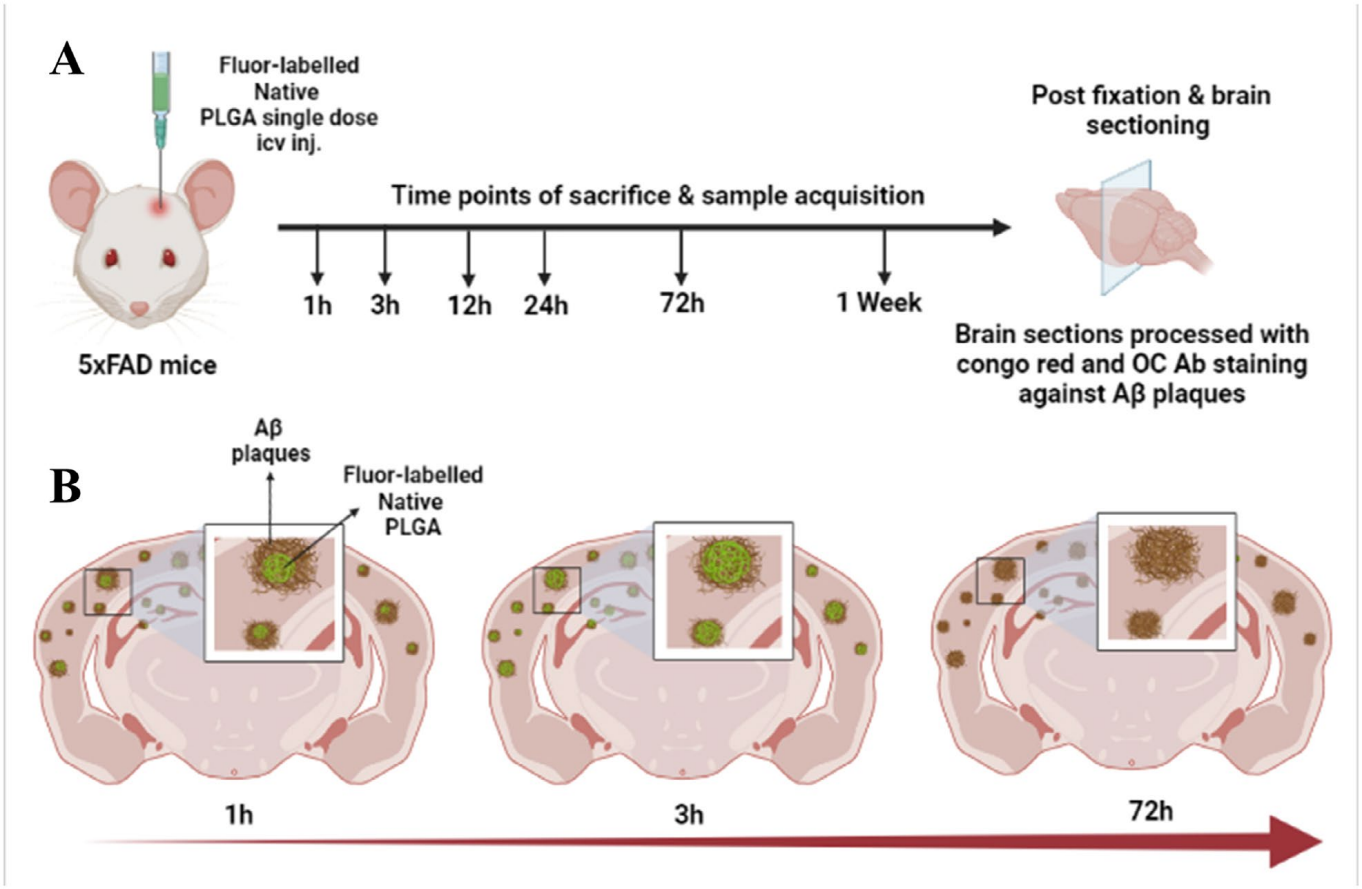
Schematic of experimental design and obtained results. **A**; Schematic representation depicting timeline of the intracerebroventricular administration of fluorescent labelled native PLGA into the brains of 5xFAD and wildtype control mice and subsequent procedure. **B**; Graphical illustration showing the observed colocalization of fluorescently labelled PLGA with extracellular neuritic plaques in the brains of 5xFAD mice at different time points (created by BioRender.com)

Six-month old 5xFAD along with age-matched WT control mice (n = 3/group) were fixed to a stereotaxic apparatus under anesthesia and a hole was drilled in the skull at the ventricular co-ordinates (-0.8 mm mid/lateral, -0.1 mm antero/posterior from Bregma). A Hamilton syringe containing green fluorescence labelled native PLGA (25 µM) was inserted into the ventricular pocket (-2.5 mm dorso/ventral from Bregma) and a single dose of 5 µl PLGA was slowly administered [26]. The fluorescence labelled PLGA injected mice were euthanized 1 h, 3 h, 12 h, 24 h, 72 h and 1-week post-injections and their brains were post-fixed in 4% paraformaldehyde for further processing. A schematic representation of the experimental design and obtained results were depicted in Fig. 1A and B [Created with BioRender.com].

### Immunohistochemistry and imaging

The fixed brains were sectioned to 30 μm thickness using a cryostat and the resulting cortical, hippocampal, and cerebellar sections were processed for Aβ immunostaining. In brief, the sections were treated with primary anti-Aβ OC antibody (1:2000) overnight at 4 °C followed by incubation of secondary Alexa fluor 594 (red)-conjugated antibody on the next day. The brain sections were then washed and mounted with ProLong™ gold antifade reagent and imaged at 10x, 20x and 40x magnifications using a Nikon Eclipse 90i fluorescence microscope equipped with a Retiga 2000R Q imaging system (Nikon Instruments Inc., NY, USA).

### Congo-red staining and imaging

The fluorescence labelled native PLGA injected brains sections from the cortical, hippocampal, and cerebellar regions were processed for Congo-red staining as described earlier. In brief, the free-floating sections were rinsed in distilled water for 30 s and then incubated in 0.5% Congo-red solution for 10 min. The sections were then washed three times in tap water for 30 s each and subsequently incubated in 50mM NaOH for post-quenching the autofluorescence for 30 s [27]. The sections were then washed three times in tap water, air-dried on a slide warmer for 5 min, mounted with ProLong™ gold antifade reagent and imaged at 10x and 20x magnifications using a Nikon Eclipse 90i fluorescence microscope equipped with a Retiga 2000R Q imaging system (Nikon Instruments Inc., NY, USA).

### Image analysis and colocalization coefficient analysis

The micrographs obtained at 20x magnification from the 5xFAD mice brain injected with green fluorescence labelled native PLGA counter stained with anti-Aβ OC antibody and Congo red staining were considered for the analysis. All the images with their corresponding red and green channel were imported to Image J software, converted to 8 bits with default aspect ratio and threshold settings and then subjected to a pre-installed “co-localization finder” plugin in the software. This plugin calculates a variety of colocalization parameters such as the Pearson coefficient, Manders co-efficient and other pixel-intensity-correlation measurements [28]. In addition, this plugin provides a scatter plot-2D intensity histogram with each color spot represents the value of the individual pixel in red channel image which is plotted against the value of the equivalent pixel in the green channel of the same image. The frequency of the intensity is coded by the color/brightness in the scatter plot. On analyzing these plots using an imaginary slope line - the graph exhibiting a positive slope (i.e., scatter plot proximal to the X-axis = 0 and Y-axis = 0) indicates correlation, while a zero slope (scatter plot proximal to either X axis or Y axis) suggests that the images are not correlated [29, 30]. The integrated fluorescence density of each image in both red and green channels was also quantified individually using the Image J software. The graphical representations of the Pearson correlation coefficient (Pearson’s r) values and integrated fluorescence density over time and their corresponding scatter plots were described in the results section.

### Statistical analysis

All statistical analyses were performed using GraphPad Prism (GraphPad Software, Inc., CA, USA). All data are presented as the mean ± standard error of the mean (S.E.M.). The mean values were determined using quantitative image analysis of four distinct loci from two brain sections of three individual animals per time point. Differences in the mean Rr values at 1 week compared to other time points (1 h, 3 h, 12 h, 24 and 72 h) were assessed by one-way analysis of variance (ANOVA) using Dunnett’s multiple comparisons post-hoc tests. A *p*-value of less than 0.05 was accepted as statistically significant.

## Results

### FITC-labelled native PLGA colocalizes with Aβ-containing neuritic plaques in 5xFAD mice

**Fig. 2:**
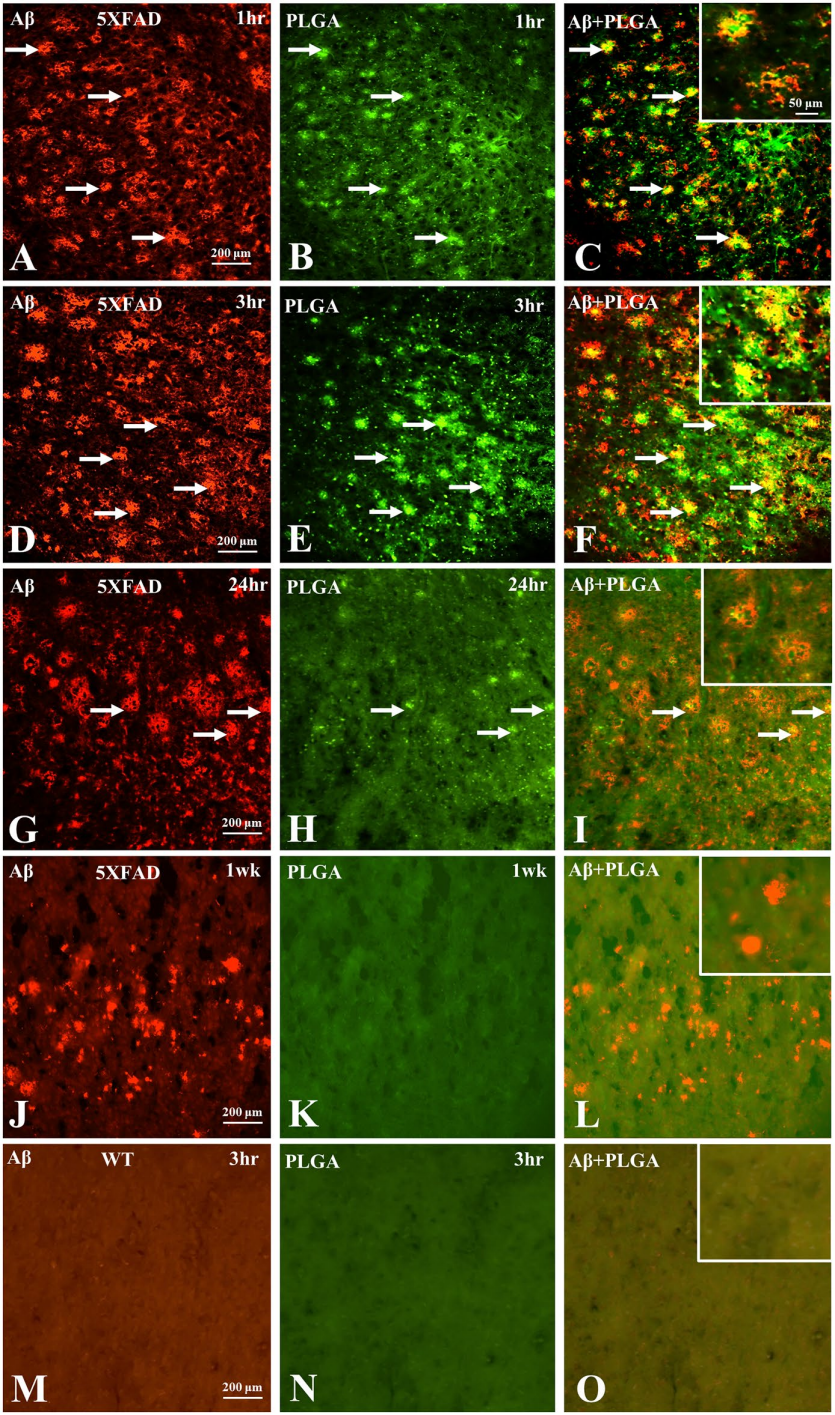
Images of neuritic plaques stained with OC antibody and labelled PLGA in the cortex. **A-L**; Photomicrographs of the cortex of 5xFAD mouse brains depicting localization of immunoreactive Aβ_1-42_ (red; **A, D, G, J**), fluorescent labelled native PLGA (green; **B, E, H, K**) and their co-localization (arrows; **C, F, I, L**) at 1 h (**A-C**), 3 h (**D-F**), 24 h (**G-I**) and 1 week (**J- L**) following acute administration of labelled PLGA into the brain. Note the colocalization (arrows) of fluorescent labelled native PLGA with Aβ-positive neuritic plaques labelled with OC antibody and the gradual decline of labelled PLGA with time in 5xFAD mouse brains. **M-O**; Photomicrographs of the cortex of wild-type (WT) control mice depicting lack of immunoreactive Aβ_1-42_ (**M**), fluorescent labelled native PLGA (**N**) and their co-localization (**O**) at 3 h following acute administration of labelled PLGA into the brain

**Fig. 3:**
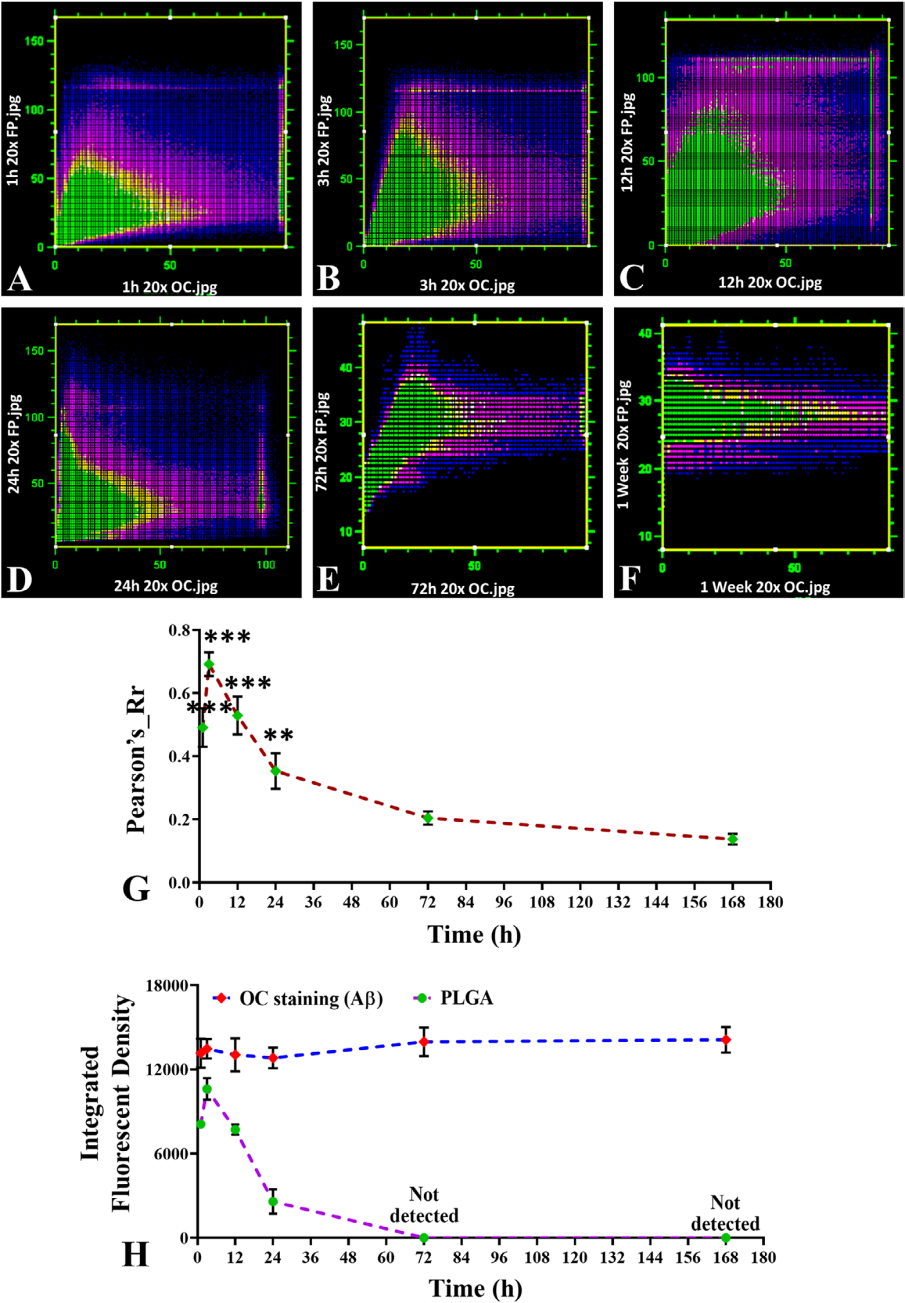
Co-efficient analysis of neuritic plaques stained with OC antibody and labelled PLGA in the cortex. **A-G**; Image co-localization analysis demonstrated by scatter plots (**A-F**) and Pearson’s co-efficient-Rr values (**G**) for the merged images of native PLGA labelled amyloid plaques in 5xFAD brain cortical tissues as a function of time. In the scatter plot, green signal intensity of native PLGA is represented on the y-axis and red pixel intensity of OC antibody labelled Aβ immunoreactivity is represented on the x-axis. A Pearson’s co-efficient-Rr value of 1 represents perfect colocalization, while 0 represents no colocalization. Note the level of colocalization as a function of time (**A-F**) reflected in the Pearson’s co-efficient graph with a max peak Rr value of 0.69 ± 0.07 at 3 h (**G**). Differences in the mean Rr values at 1 week compared to other time points (1 h, 3 h, 12 h, 24 and 72 h) were assessed by one-way ANOVA employing Dunnett’s multiple comparisons post-hoc tests. * *p* < 0.05; ** *p* < 0.01 and *** *p* < 0.001. **H**; Graph representing integrated fluorescence densities of labelled PLGA (lavender) and OC antibody labelled Aβ-containing plaques (blue) from 5xFAD mouse brain images depicting a decline in fluorescence densities of PLGA, but not OC antibody, as a function of time over 72hr period

Recently we reported that chronic icv administration of unlabelled native PLGA using miniosmotic pump attenuates non-spatial object memory deficits and reduces markedly the number of Aβ plaques in the affected cortical regions of 5xFAD mice compared to CSF-treated 5xFAD mice. This could be due to inhibition of the spontaneous aggregation and/or disassembly of aggregated Aβ peptides. Furthermore, this is accompanied by a decreased level of Aβ_1−40_/Aβ_1−42_ as well as the levels of APP holoprotein and α-/β-CTFs in the cortex of PLGA-treated 5xFAD mice compared to CSF-treated 5xFAD mice. Unlike cortex, PLGA did not significantly alter the levels of APP, α-/β-CTFs or Aβ_1−40_/Aβ_1−42_ either in the cerebellum of 5xFAD mice or in any brain regions of WT control mice, suggesting that the effects may be specific to the affected areas associated with increased levels/deposition of Aβ peptides [23]. Given the evidence that native PLGA can bind Aβ under in vitro conditions [23, 26], we wanted to determine if PLGA can recognize Aβ-containing neuritic plaques in 5xFAD mouse brains. Prior to administering labelled PLGA into the brain, we revealed that FITC-labelled PLGA nanoparticles displayed hydrodynamic radii of ~ 100 nm, a Zeta potential of -17.4 mV and PDI of 0.198 (Supplementary Fig. S1). After that, we acutely administered FITC-labelled PLGA into the brain of 5xFAD and age-matched WT control mice and evaluated localization of labelled PLGA with immunoreactive Aβ peptide at different time points (Figs. 2 and 3; Supplementary Fig. S2). Our results clearly show that fluorescently labelled native PLGA following acute intracerebellar injection interacts with extracellular Aβ-containing neuritic plaques immunolabelled with aggregate specific OC antibody in 5xFAD mice (Fig. 2A-I; Fig. Supplementary S2A-C). This is evident at 1 h (Fig. 2A-C), peak around 3 h (Fig. 2D-F) and then start declining by 24 h (Fig. 2G-I) after injection. Interestingly, native PLGA was found to be localized mostly in the center of the neuritic plaques, though some are also distributed in the plaque periphery.

**Fig. 4:**
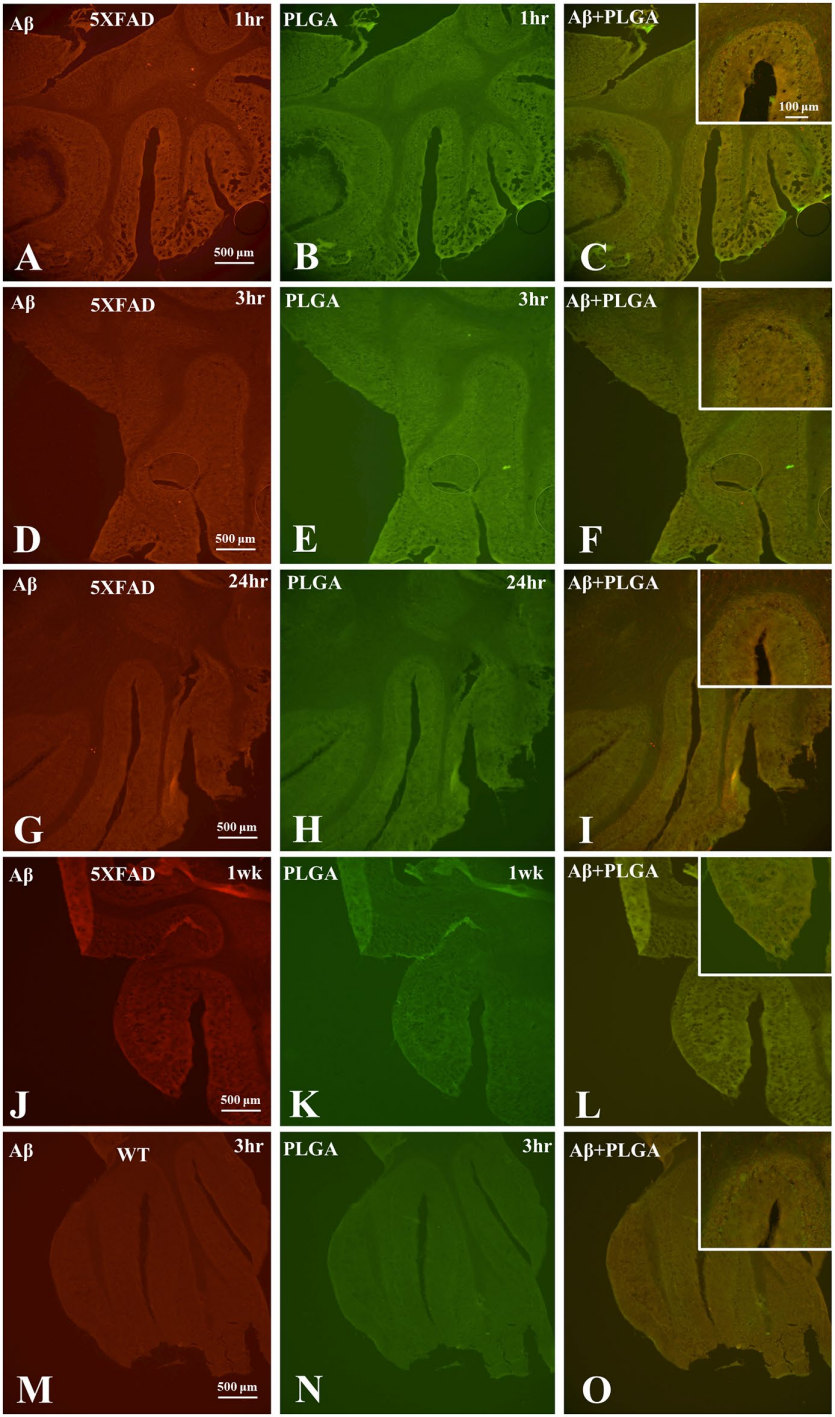
Images of neuritic plaques stained with OC antibody and labelled PLGA in the cerebellum. **A-L**; Photomicrographs of the cerebellum of 5xFAD mice depicting localization of immunoreactive Aβ_1−42_ (red; **A, D, G, J**), fluorescent labelled native PLGA (green; **B, E, H, K**) and their co-localization (arrows; **C, F, I, L**) at 1 h (**A-C**), 3 h (**D-F**), 24 h (**G-I**) and 1 week (**J- L**) following acute administration of labelled PLGA into the brain. Note the lack of immunoreactive Aβ-positive neuritic plaques, fluorescent labelled native PLGA and their colocalization in the cerebellum over the course of experimental timeline in 5xFAD mouse brains. **M-O**; Photomicrographs of the cerebellum of wild-type (WT) control mice depicting lack of immunoreactive Aβ_1−42_ (**M**), fluorescent labelled native PLGA (**N**) and their co-localization (**O**) at 3 h following acute administration of labelled PLGA into the brain

As apparent from our double labelling experiments, majority of Aβ-containing neuritic plaques exhibit PLGA labelling thus validating the specificity of interaction. This was clearly depicted in the scatter plot-2D intensity histograms and corresponding graphs representing Pearson correlation coefficient (Pearson’s r) values revealing the interaction timeline of labelled PLGA with OC antibody labelled amyloid plaques (Fig. 3A-G). When the intensities of two different colour channels (i.e., labelled PLGA and OC immunolabelling) display a merged scatter plot pattern exactly at the zero-zero of x- and y-axes in the scattered plot and the Pearson’s “r” value close to + 1 indicates high colocalization [31]. From 1 to 24 h the colocalization pattern was close to the zero for both the axes (Fig. 3A-G), at 3h a high colocalization was observed with Pearson’s “r” values 0.69 ± 0.07 (Fig. 3B, G), where as at 72 h and 1-week poor co-localization was observed with Pearson’s “r” values of 0.21 ± 0.02 and 0.13 ± 0.02 respectively (Fig. 3E-G), indicating the absence of labelled PLGA in the existing amyloid plaques. Further, to confirm the pattern of interaction between PLGA and extracellular neuritic plaques over time, we separately quantified the integrated fluorescence densities of labelled PLGA (i.e., green channel) and OC antibody labelled amyloid plaques (i.e., red channel) from images of 5xFAD mouse brains (Fig. 3H). Our results show that labelled PLGA, as observed with colocalization results, exhibits a similar pattern of decline i.e., fluorescence density peak at 3 h (10606.80 ± 1349) and then decrease gradually from 12 h (7713.73 ± 643) onwards with no signal evident either at 72 h or 1 week. In contrast, the OC antibody labelled amyloid plaques, as expected, showed consistent fluorescence density (13429.10 ± 1453) in the 5xFAD cortical brain region. In contrast to the cortical region of 5xFAD mice, the fluorescently labelled PLGA did not accumulate in the cerebellum of 5xFAD (Fig. 4A-O; Supplementary Fig. S2G-L) and overall regions of brain in control mice (Fig. 2M-O) at any time following intracerebellar injection.

### FITC-labelled native PLGA colocalizes with Congo red labelled neuritic plaques in 5xFAD mice

**Fig. 5:**
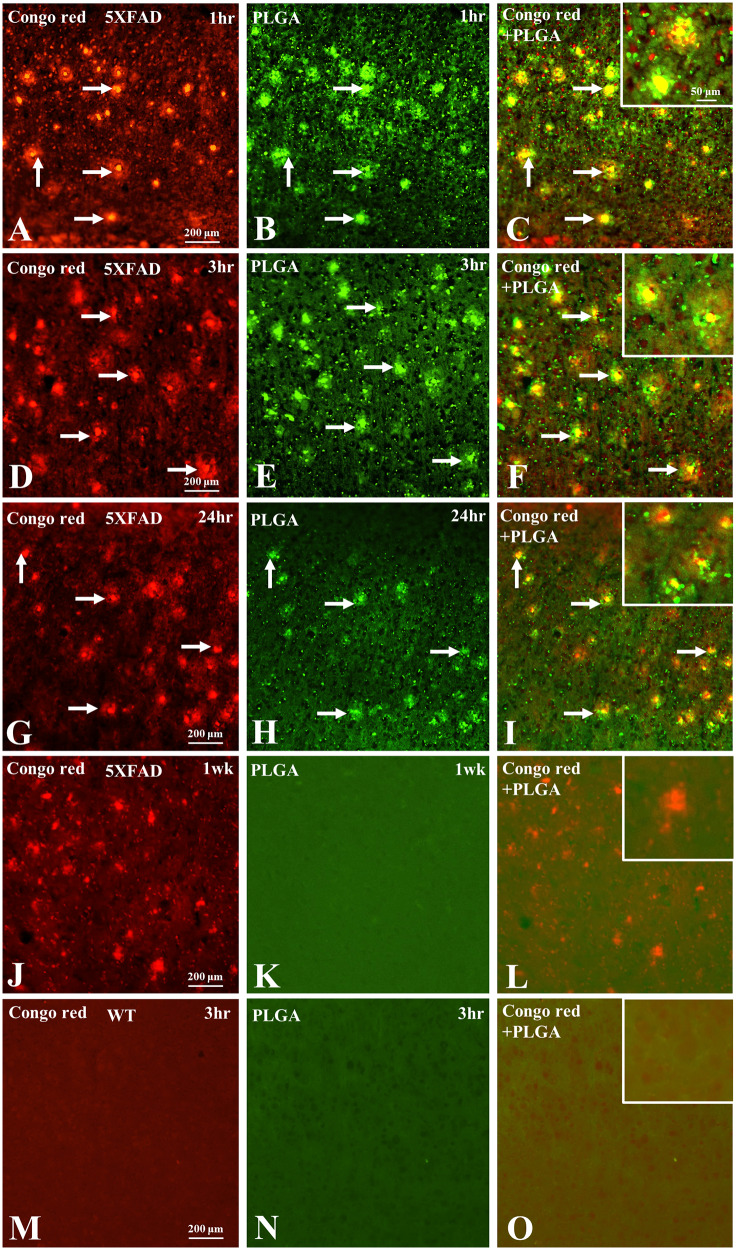
Images of neuritic plaques stained with Congo Red and labelled PLGA in the cortex. **A-L**; Photomicrographs of the cortex of 5xFAD mouse brains depicting localization of Congo Red (red; **A, D, G, J**), fluorescent labelled native PLGA (green; **B, E, H, K**) and their co-localization (arrows; **C, F, I, L**) at 1 h (**A-C**), 3 h (**D-F**), 24 h (**G-I**) and 1 week (**J- L**) following acute administration of labelled PLGA into the brain. Note the colocalization (arrows) of fluorescent labelled native PLGA with Congo Red-positive neuritic plaques and the gradual decline of labelled PLGA with time in 5xFAD mouse brains. **M-O**; Photomicrographs of the cortex of wild-type (WT) control mice depicting lack of Congo Red (**M**), fluorescent labelled native PLGA (**N**) and their co-localization (**O**) at 3 h following acute administration of labelled PLGA into the brain

**Fig. 6:**
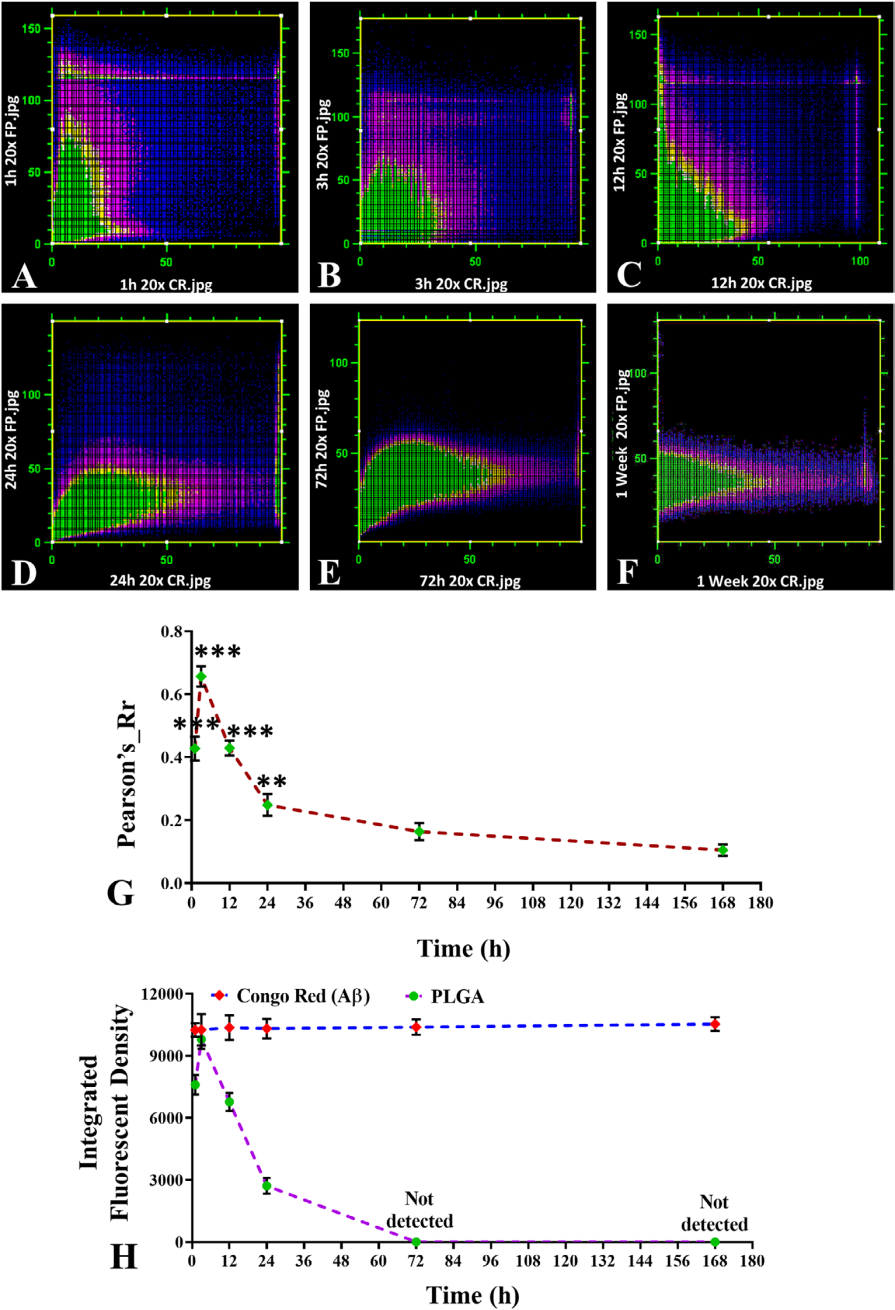
Co-efficient analysis of neuritic plaques stained with Congo Red and labelled PLGA in the cortex. **A-G**; Image co-localization analysis demonstrated by scatter plots (**A-F**) and Pearson’s co-efficient-Rr values (**G**) for the merged images of native PLGA labelled amyloid plaques in 5xFAD brain cortical tissues as a function of time. In the scatter plot, green signal intensity of native PLGA is represented on the y-axis and red pixel intensity of Congo Red labelled plaques is represented on the x-axis. A Pearson’s co-efficient-Rr value of 1 represents perfect colocalization, while 0 represents no colocalization. Note the level of colocalization as a function of time (**A-F**) reflected in the Pearson’s co-efficient graph with a max peak Rr value of 0.67 ± 0.05 at 3 h (**G**). Differences in the mean Rr values at 1 week compared to other time points (1 h, 3 h, 12 h, 24 and 72 h) were assessed by one-way ANOVA employing Dunnett’s multiple comparisons post-hoc tests. * *p* < 0.05; **p < 0.01 and ***p < 0.001 **H**; Graph representing integrated fluorescence densities of labelled PLGA (lavender) and Congo Red labelled plaques (blue) from 5xFAD mouse brain images depicting a decline in fluorescence densities of PLGA, but not Congo Red, as a function of time over 72hr period

It is well established that Congo red dye is commonly used in the histological staining of amyloid plaques in the brains of both the animal models of AD as well as post-mortem human AD patients which appear as apple-green birefringence in polarized light and bright red color fluorescence in Texas red filter (i.e., 561-590 nm) [27]. To validate that FITC-labelled PLGA detect Aβ-containing neuritic plaques, we performed Congo-red staining of brain sections of 5xFAD mice at different times following single intracerebral injection of labelled PLGA. Our results clearly indicate that fluorescently labelled native PLGA colocalized with Congo-red labelled Aβ-containing neuritic plaques (Figs. 5 and 6; Supplementary Fig. S3). Interestingly, as observed with OC immunolabelling, neuritic plaques labelled with Congo-red and fluorescent PLGA were evident at 1 h (Fig. 5A-C), peak around 3 h (Fig. 5D-F) and then declined by 24 h (Fig. 5G-I) following single intracerebral injection. This was depicted in the scatter plot-2D intensity histograms and corresponding graphs representing Pearson’s “r” values obtained as a function of time (Fig. 6A-G). The Pearson’s “r” value was 0.40 ± 0.04 at 1 h, increased gradually to reach 0.67 ± 0.05 at 3 h and then declined as a function of time to reach plateau of 0.10 ± 0.03 over 1 week (Fig. 6G) following intracerebral administration. This was further confirmed by the quantifications of the integrated fluorescence densities of Congo-red stained amyloid plaques (i.e., red channel) and PLGA (i.e., green channel) from 5xFAD mouse brain images (Fig. 6H). As expected, labelled PLGA showed a peak fluorescence density at 3 h (9795.06 ± 469) and then start declining from 12 h (6772.89 ± 436) onwards, while no signal was detected at 72 h or 1 week after administration. Conversely, Congo-red labelled plaques did not decrease at any time in the 5xFAD mouse brain. Unlike the cortical region of 5xFAD mice, the fluorescently labelled PLGA did not accumulate in the cerebellum of 5xFAD (Supplementary Fig. S3G-L) and overall regions of brain in control mice (Fig. 4M-O) at any time following intracerebellar injection.

## Discussion

The present study revealed that fluorescent labelled native PLGA nanoparticles following acute administration can recognize extracellular Aβ deposition in the brain of 5xFAD mice, thus raising its potential use in the diagnosis of AD. This is supported by results which show that: (i) labelled native PLGA can identify majority of immunostained Aβ- as well as Congo red-labelled extracellular neuritic plaques in the cortical regions of 5xFAD mice, (ii) PLGA-labelled plaques were apparent at 1 h following injection, peak around 3 h and then start declining from 24 h onwards and (iii) fluorescent labelled PLGA was not detected in the unaffected cerebellum of 5xFAD mice or in any brain regions of wild-type control mice at any time following administration. These results suggest that native PLGA nanoparticles, which have previously shown to attenuate AD-related cognitive deficits as well as pathology, may have unique potential in tracking in vivo pathological changes in AD pathology.

Imaging biomarkers for pathologic proteins involve in AD pathogenesis are vital for clinical diagnosis, disease staging and monitoring of the potential therapeutic approaches. Since extracellular Aβ deposition occurs ~ 10–20 years prior to the onset of typical AD symptoms [10, 32], non-invasive visualization of Aβ plaques using PET, single-photon emission computerized tomography (SPECT) or MRI is being actively investigated to advance the clinical landscape of AD diagnosis [4, 11]. At present, however, PET imaging is used more commonly than others due to the availability multiple clinically approved radiolabelled tracers to image Aβ aggregates in AD brains. It has been suggested that Aβ PET imaging, together with comprehensive clinical and cognitive assessment, increases etiological diagnosis of AD in ~ 25–31% of cases and patient management in ~ 37–72% of cases [4, 10]. Apart from the widely used ^11^ C-PIB, three ^18^ F-labelled PET tracers approved for clinical use include ^18^ F-Florbetaben, ^18^ F-Flutemetamol and ^18^ F-Florbetapir – all of which have specific affinity for both neuritic and diffuse plaques as well as amyloid-laden blood vessels in cerebral amyloid angiopathy [33, 34]. Each of these tracers which interact noncovalently with hydrophobic pockets/channels present within Aβ aggregates exhibit different pharmacokinetics, chemical structure and binding site/properties [4, 35]. While short half-life (20 min) of ^11^ C-labelled tracer (i.e., ^11^ C-PIB) limits its availability to centers with on-site cyclotron and radiochemistry expertise, ^18^ F-labelled tracers with a half-life of 110 min can be produced centrally and used more widely [10, 36]. Although these Aβ tracers have provided valuable information on the load/distribution of Aβ-containing plaques in AD pathology, relatively high levels of white matter retention due to nonspecific binding limit their signal detection sensitivity. High sensitivity is of interest especially in prodromal phases of AD when plaque load is relatively low [9]. Interestingly, with the advancements of various imaging techniques, multimodal imaging (i.e., PET/MRI; MRI/SPECT; PET/CT; structural/functional MRI) modalities are gaining momentum in AD diagnosis to obtain precise details and enhanced spatial resolution at specific brain sites for comprehensive assessment [37, 38]. Thus, extensive research is currently being carried out to develop new tracers that can not only overcome the limitations of previous agents, i.e., safety, efficacy, non-specific binding and rather short half-lives, but also can assist in emerging therapies as well as in the progression of disease pathology.

In recent years, apart from the clinically approved imaging tracers to detect Aβ aggregates, various nanoparticles, as observed in the present study, have shown promising nano-diagnostic opportunity in the early detection of AD. For example, it was demonstrated that Fe bio-metal accumulates specifically in amyloid plaque core as crystalline magnetic nanoparticles and has superparamagnetic properties that can be used in AD diagnosis [39]. Additionally, nanocarriers loaded with imaging agents such as curcumin or Aβ antibodies have been developed to image and/or monitor Aβ in the brains [40–42]. Interestingly, our study reveals that FITC labelled native PLGA nanoparticles, in keeping with the diagnostic ability of some functionalized nanoparticles, can detect in vivo Aβ aggregates around 1 h (the time first evaluated) following *icv* administration. The colocalization of PLGA with Aβ-containing neuritic plaques peak around 3 h and then start declining gradually possibly due to dissociation of FITC or breakdown of PLGA polymer. At present, precise mechanism by which labelled PLGA interacts with neuritic plaques remains unclear. We have recently reported that native PLGA can interact with the hydrophobic domain (i.e., Lys_16_ to Ala_21_) of Aβ_1−42_ which may underlie its ability to attenuate Aβ aggregation [23, 26]. Additionally, an interface of PLGA with residues located in the steric zipper domain of Aβ, as observed with a variety of phytochemicals and other molecules [43, 44], may trigger disassembly of preformed Aβ aggregates [23]. Thus, it is likely that labelled PLGA due to its intrinsic ability to interact with the hydrophobic and other domains of Aβ peptides can recognize extracellular neuritic plaques in 5xFAD mouse brain. This is consistent with some recent studies demonstrating the presence of Aβ-containing neuritic plaques in mutant APP double transgenic mice as well as 5xFAD mice using a novel phenothiazine derivative PHTZ-1 and a fluorophore DBAN-SLM, respectively [45, 46]. It is also worth noting that the majority, but not all, extracellular neuritic plaques recognized by Aβ antibody or Congo red, were identified with labelled PLGA. Whether it is the consequence of the employed dose of the labelled PLGA and/or the time of imaging following nanoparticle administration remains to be determined. Additionally, it will be of interest to evaluate not only the site but also the mechanism underlying the interaction between native PLGA and Aβ aggregates located in the neuritic plaques in 5xFAD mouse brains. Despite these limitations, our results provide a proof of principle that tracer-/probe-labelled PLGA may have the potential, if can be designed to cross the BBB, in the diagnosis of AD pathology.

Some earlier studies have shown that PLGA nanoparticles conjugated with potential drug/agent when functionalized at the surface with molecules such as B6 peptide, Tet-1 peptide, vitamin D or Tween 80 etc. are able to cross the protective BBB [47–52]. Thus, if labelled native PLGA can be surface functionalized similarly with any of the aforesaid molecules, it will enable the labelled PLGA to cross BBB and recognize Aβ-containing neuritic plaques in AD brains. Additionally, it is of interest to note that most of the PET tracers currently in use for the diagnosis of AD are administered intravenously [10, 11]. Since PLGA nanoparticles when conjugated with some potential drugs/agents have been shown to exhibit beneficial effects on AD-related pathology following intravenous administration [51, 52], it may be feasible to develop ^18^F-labelled PLGA nanoparticles that can be delivered intravenously to detect the presence of Aβ-containing neuritic plaques for the diagnosis and/or disease staging of AD pathology. In fact, an earlier study using an ^18^ F-labelled radioligand attached to avidin modified PLGA nanoparticles have been tested by PET imaging to measure the kinetics and spatial volume of distribution following delivery to the rat brain. These ^18^ F-labelled PLGA nanoparticles were found to be well tolerated without any adverse reactions to the animals. Interestingly, in recent years several studies have employed PLGA as a nano-carrier to transport probes to distinct brain regions for diagnostic purposes using MRI and PET imaging [53, 54]. Thus, it will be of future interest to determine if PLGA nanoparticles tagged with fluorophores and/or various PET/SPECT/MRI probes can serve as a novel multimodal imaging agent for detecting Aβ aggregates with high relatively high affinity and specificity in established animal models of AD as well as in human AD patients.

Over the last decade several studies have shown that PLGA nanoparticles when conjugated/encapsulated with donepezil, galantamine, and curcumin can exhibit beneficial effects on cellular and/or animal models of AD [50, 55–57]. Nevertheless, an earlier study first reported that native PLGA without conjugation with any drug/agent can attenuate the death of cultured cells caused by the mitochondrial toxin 1-methyl-4-phenyl-1,2,3,6-tetrahydropyridine (i.e., MPTP - used in the development of an animal model of Parkinson Disease, PD) or genetic mutation associated with PD. Intracerebral injection of PLGA was also found to attenuate MPTP-related neurodegeneration and pathology [58]. More recently, we reported that native PLGA can ameliorate not only Aβ aggregation/toxicity but also AD-related pathology in cellular and animal models of AD [23, 24]. This is supported by results showing that (i) PLGA inhibits spontaneous Aβ aggregation and triggers the disassembly of matured Aβ_1−42_ fibers, (ii) PLGA treatment protects mouse primary neurons against Aβ-mediated toxicity by reducing phosphorylation of tau protein and reducing lysosomal pH, (iii) chronic icv administration of PLGA using Alzet miniosmotic pump reverses cognitive deficits and attenuates Aβ levels/plaque load in 5xFAD mice and (iv) PLGA protects induced pluripotent stem cells **(**iPSC)-derived neurons of AD patients against Aβ toxicity by decreasing phosphorylation of tau protein. Collectively, these results suggest that native PLGA may have unique therapeutic potential in the treatment of AD pathology.

## Conclusion

The present study provides evidence that labelled native PLGA nanoparticles without conjugation with any drug/agent can be able to detect in vivo Aβ deposition in an established model of AD. These results, together with our earlier data, raise the possibility that native PLGA may have potential to serve as a novel nano-theragnostic agent representing an integrated strategy for the diagnosis as well as treatment of AD pathology.

### Electronic supplementary material

Below is the link to the electronic supplementary material.

Supplementary Material 1: **Suppl. Figure 1. Characterization of Fluorescence labelled PLGA A-C**; DLS analysis of labelled PLGA nanoparticles depict a peak of ~100nm average diameter (A), Zeta potential displaying surface charge -17.4mV (B) and its corresponding value with polydispersity index of 0.198 are presented in the Table (C).

Supplementary Material 2: **Suppl. Figure 2. Images of Aβ immunoreactivity and labelled PLGA in the cortex and cerebellum A-F**; Photomicrographs of the cortex of 5xFAD mouse brains depicting localization of immunoreactive Aβ_1-42_ (red; A, D), fluorescent labelled native PLGA (green; B, E) and their co-localization (arrows, C, F) at 12hr (A-C) and 72hr (D-F) following acute administration of labelled PLGA into the brain. Note the colocalization (arrows) of fluorescent labelled native PLGA with A-positive neuritic plaques labelled with OC antibody at 12hr and the decline of labelled PLGA at 72hr in 5xFAD mouse brains. **G-L**; Photomicrographs of the cerebellum of 5xFAD mice depicting localization of immunoreactive Aβ_1-42_ (red; G, J), fluorescent labelled native PLGA (green; H, K) and their co-localization (I, L) at 12hr (G-I) and 72hr (J-L) following acute administration of labelled PLGA into the brain. Note the lack of immunoreactive Aβ-positive neuritic plaques, fluorescent labelled native PLGA and their colocalization in the cerebellum at either 12hr or 72hr in 5xFAD mouse brains.

Supplementary Material 3: **Suppl. Figure 3. Images of Congo Red and labelled PLGA in the cortex and cerebellum A-F**; Photomicrographs of the cortex of 5xFAD mouse brains depicting localization of Congo Red (red; A, D), fluorescent labelled native PLGA (green; B, E) and their co-localization (arrows, C, F) at 12hr (A-C) and 72hr (D-F) following acute administration of labelled PLGA into the brain. Note the colocalization (arrows) of fluorescent labelled native PLGA with Congo Red-positive neuritic plaques at 12hr and the decline of labelled PLGA at 72hr in 5xFAD mouse brains. **G-L**; Photomicrographs of the cerebellum of 5xFAD mice depicting localization of Congo Red (red; G, J), fluorescent labelled native PLGA (green; H, K) and their co-localization (I, L) at 12hr (G-I) and 72hr (J-L) following acute administration of labelled PLGA into the brain. Note the lack of Congo Red-positive neuritic plaques, fluorescent labelled native PLGA and their colocalization in the cerebellum at either 12hr or 72hr in 5xFAD mouse brains.

## Data Availability

The data in this work are available in the manuscript or Supplementary Information, or available from the corresponding author upon reasonable request.

## Abbreviations

Aβ: Amyloid β
AD: Alzheimer’s disease
BBB: Blood-brain barrier
APP: Amyloid precursor protein
CSF: Cerebrospinal fluid
FDA: US Food and Drug Administration
FITC: Fluorescein isothiocyanate
MRI: Magnetic resonance imaging
PDI: Polydispersity index
PET: Positron emission tomography
PiB: Pittsburgh Compound-B
PS1: Presenilin 1
PLGA: Poly(D,L-lactide-co-glycolide)
SPECT: Single-photon emission computerized tomography
WT: Wild-type
icv: Intracerebroventricular
MPTP: 1-methyl-4-phenyl-1,2,3,6-tetrahydropyridine
