# Correction: Detection of β-amyloid aggregates/plaques in 5xFAD mice by labelled native PLGA nanoparticles: implication in the diagnosis of Alzheimer’s disease

**DOI:** 10.1186/s12951-023-02009-8

**Published:** 2023-08-03

**Authors:** Karthivashan Govindarajan, Satyabrata Kar

**Affiliations:** Departments of Medicine (Neurology), Centre for Prions and Protein Folding Diseases, Neuroscience and Mental Health Institute, University of Alberta, Edmonton, AB T6G 2M8 Canada

**Correction: Journal of Nanobiotechnology (2023) 21:216**

10.1186/s12951-023-01957-5

Following publication of the original article [1], the authors identified an error in the figure citation. The change is highlighted in **bold typeface.**

“Pearson’s “r” values obtained as a function of time (Fig. 5A-G)” should be replaced with “Pearson’s “r” values obtained as a function of time **(Fig. 6A-G)”.**

“The original article [1] has been corrected”.
