# Supplementary figures and images for: Detection of β-amyloid aggregates/plaques in 5xFAD mice by labelled native PLGA nanoparticles: implication in the diagnosis of Alzheimer’s disease
Source: J Nanobiotechnology. 2023 Jul 10;21:216. doi: 10.1186/s12951-023-01957-5 (PMC10332042; doi:10.1186/s12951-023-01957-5)

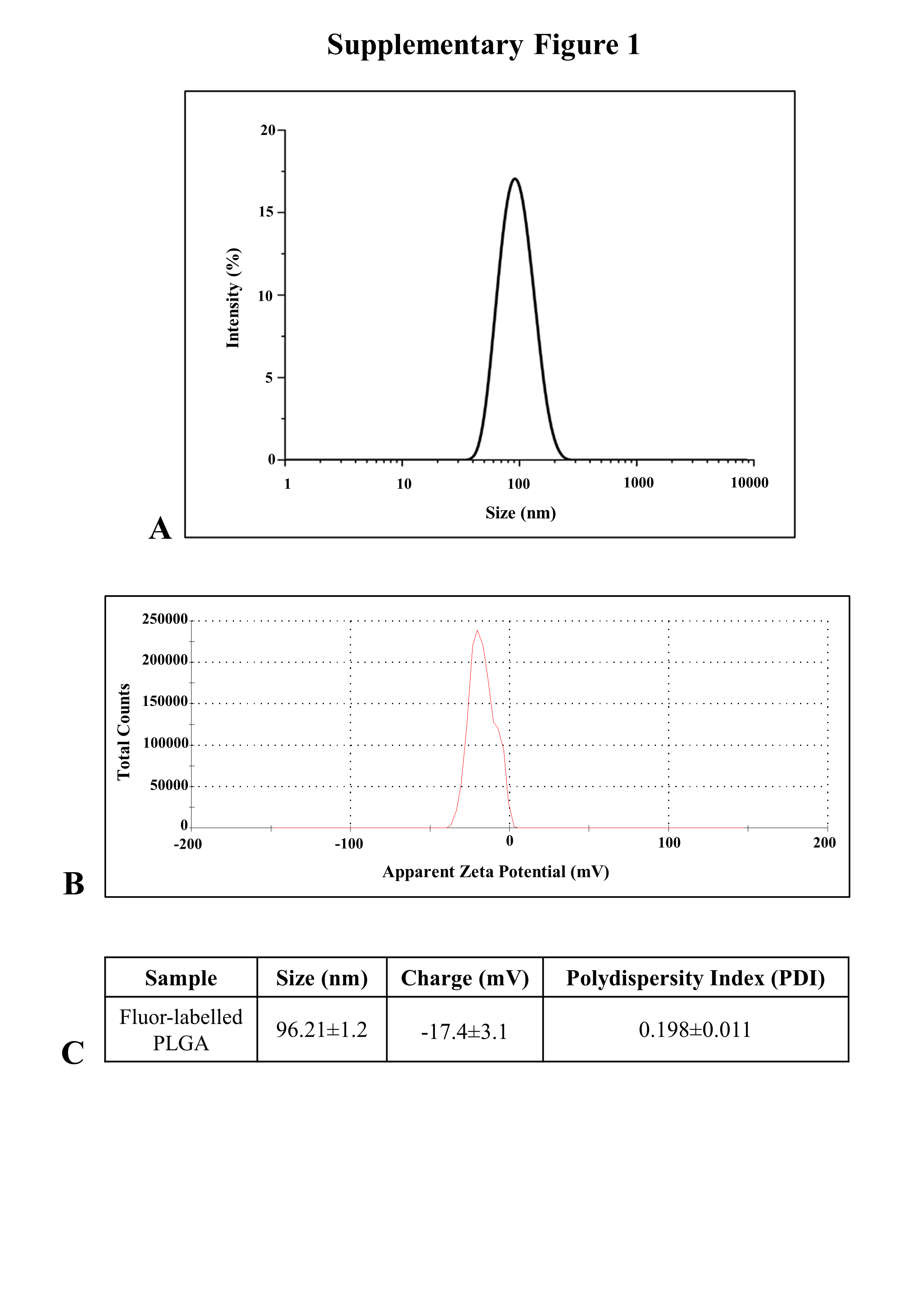

Supplement: Supplementary file 1 — Supplementary Material 1: Suppl. Figure 1. Characterization of Fluorescence labelled PLGA A-C; DLS analysis of labelled PLGA nanoparticles depict a peak of ~100nm average diameter (A), Zeta potential displaying surface charge -17.4mV (B) and its corresponding value with polydispersity index of 0.198 are presented in the Table (C). [file 12951_2023_1957_MOESM1_ESM.png]

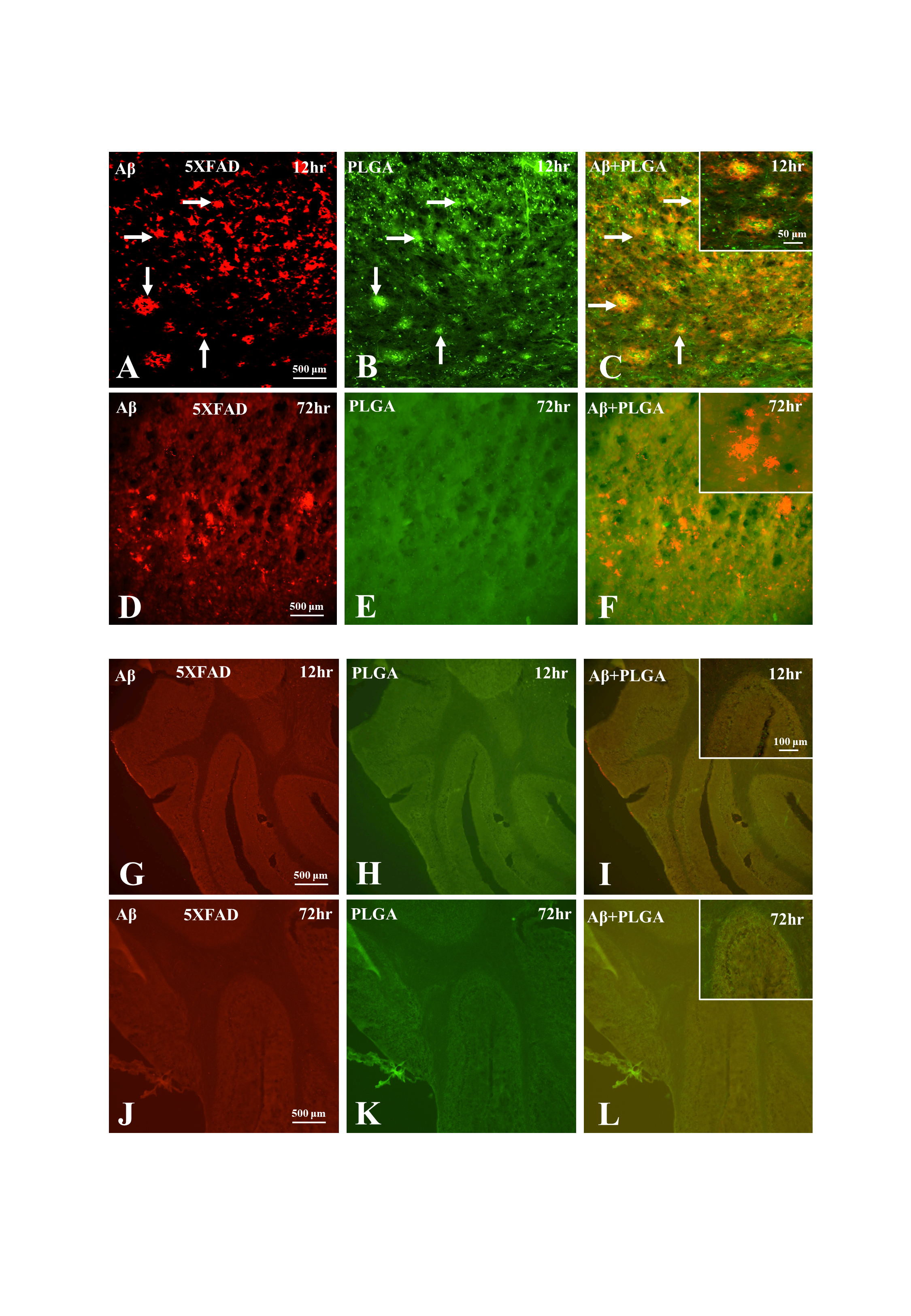

Supplement: Supplementary file 2 — Supplementary Material 2: Suppl. Figure 2. Images of Aβ immunoreactivity and labelled PLGA in the cortex and cerebellum A-F; Photomicrographs of the cortex of 5xFAD mouse brains depicting localization of immunoreactive Aβ1-42 (red; A, D), fluorescent labelled native PLGA (green; B, E) and their co-localization (arrows, C, F) at 12hr (A-C) and 72hr (D-F) following acute administration of labelled PLGA into the brain. Note the colocalization (arrows) of fluorescent labelled native PLGA with A-positive neuritic plaques labelled with OC antibody at 12hr and the decline of labelled PLGA at 72hr in 5xFAD mouse brains. G-L; Photomicrographs of the cerebellum of 5xFAD mice depicting localization of immunoreactive Aβ1-42 (red; G, J), fluorescent labelled native PLGA (green; H, K) and their co-localization (I, L) at 12hr (G-I) and 72hr (J-L) following acute administration of labelled PLGA into the brain. Note the lack of immunoreactive Aβ-positive neuritic plaques, fluorescent labelled native PLGA and their colocalization in the cerebellum at either 12hr or 72hr in 5xFAD mouse brains. [file 12951_2023_1957_MOESM2_ESM.tif]

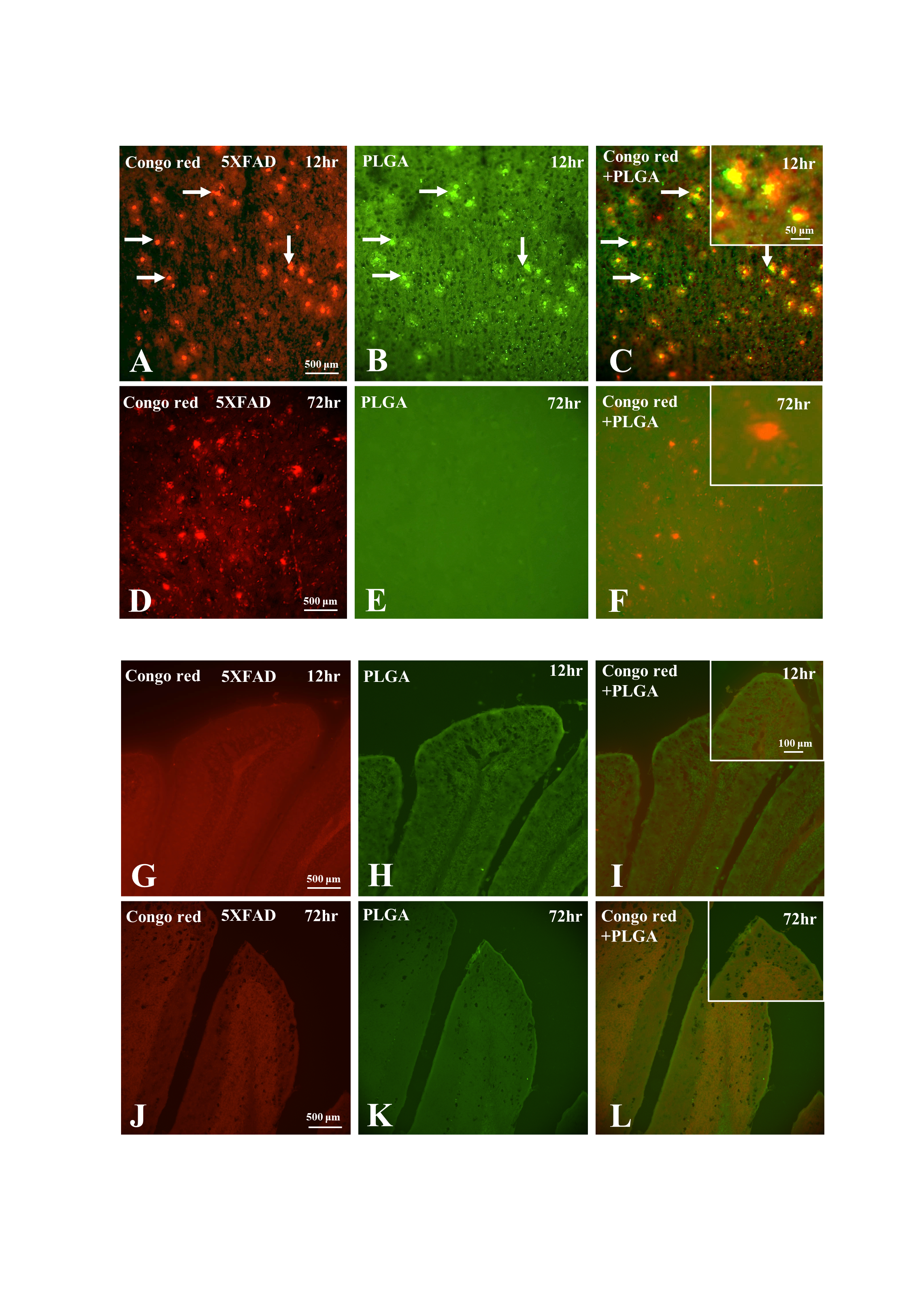

Supplement: Supplementary file 3 — Supplementary Material 3: Suppl. Figure 3. Images of Congo Red and labelled PLGA in the cortex and cerebellum A-F; Photomicrographs of the cortex of 5xFAD mouse brains depicting localization of Congo Red (red; A, D), fluorescent labelled native PLGA (green; B, E) and their co-localization (arrows, C, F) at 12hr (A-C) and 72hr (D-F) following acute administration of labelled PLGA into the brain. Note the colocalization (arrows) of fluorescent labelled native PLGA with Congo Red-positive neuritic plaques at 12hr and the decline of labelled PLGA at 72hr in 5xFAD mouse brains. G-L; Photomicrographs of the cerebellum of 5xFAD mice depicting localization of Congo Red (red; G, J), fluorescent labelled native PLGA (green; H, K) and their co-localization (I, L) at 12hr (G-I) and 72hr (J-L) following acute administration of labelled PLGA into the brain. Note the lack of Congo Red-positive neuritic plaques, fluorescent labelled native PLGA and their colocalization in the cerebellum at either 12hr or 72hr in 5xFAD mouse brains. [file 12951_2023_1957_MOESM3_ESM.tif]
